# Sustainable preservation of dried fish using hybridized plant extracts: HPLC-based quantification of aflatoxins and vitamins, and assessment of broad-spectrum antimicrobial efficacy

**DOI:** 10.1371/journal.pone.0347254

**Published:** 2026-04-24

**Authors:** Manal Almughamisi, Hilary Uguru, Dennis Adaigho, Ola A. Abu Ali, Sarah Alharthi, Rokayya Sami, Roqayah H. Kadi, Afnan M. Alnajeebi, Amera N. Alqahtani, Shatha H. Alaoufi, Moayad M. Khashoqji, Alaa H. Felemban, Monya T. Jawharji, Halah A. Hafiz, Rowida Allily, Huda Wazzan

**Affiliations:** 1 Department of Clinical Nutrition, College of Applied Medical Sciences, Taibah University, Saudi Arabia; 2 Department of Agricultural Engineering, Delta State University of Science and Technology, Ozoro, Nigeria; 3 Department of Agricultural Economics, Southern Delta University, Ozoro, Nigeria; 4 Department of Chemistry, College of Science, Taif University, Taif, Saudi Arabia; 5 Research Center of Basic Sciences, Engineering and High Altitude, Taif University, Taif, Saudi Arabia; 6 Department of Food Science and Nutrition, College of Sciences, Taif University, Taif, Saudi Arabia; 7 Department of Biological Sciences, College of Science, University of Jeddah, Jeddah, Saudi Arabia; 8 Department of Early Childhood, College of Education, Qassim University, Buraydah, Saudi Arabia; 9 Applied College, Taibah University, Madinah, Saudi Arabia; 10 Department of Clinical Nutrition, Faculty of Applied Medical Sciences, Umm Al-Qura University, Saudi Arabia; 11 Department of Food and Nutrition, Faculty of Human Sciences and Design, King Abdulaziz University, Jeddah, Saudi Arabia; Amity University Noida, INDIA

## Abstract

This research aimed to preserve the nutritional qualities of fish flesh during prolonged storage, by utilizing bioactive compounds derived from plant extracts and oils. Fish flesh (tissues) was treated with lime, ginger, turmeric, banana peel extracts, and oils – including their hybridized forms. The dried fish samples nutritional quality, and microbial population were measured using established protocols. Results obtained revealed that the treatments played significant roles, in enriching the fish nutritional quality and also inhibiting microbial growth during storage. It was observed that the treatments played a substantial role, in the preservation of the nutritional integrity of dried fish (mostly the proteins and vitamins), during the extended storage duration. Also, most of the hybridized treatments displayed synergistic mechanistic effects, which resulted in enhanced antimicrobial and nutrient retention actions. Conspicuously, the lime extract played a critical role in potentiating the treatments antimicrobial efficacy (T8 to T13). The results revealed that the control sample had higher bacterial and fungal growth at week 8; whereas, treatments T8 to T13 displayed superior antimicrobial activities. Notably, T8 sample exhibited the best antimicrobial action, recording the lowest counts – *Staphylococcus spp.* (52 CFU/g), *Salmonella spp.* (14 CFU/g), *Bacillus spp.* (3 CFU/g), *Listeria spp.* (5 CFU/g), *Aspergillus spp. (black mold)* (11 CFU/g), *Penicillium spp.* (7 CFU/g), *Aspergillus spp. (flavus group)* (37 CFU/g), and *Rhizopus spp.* (14 CFU/g). It was also observed that, most of the combined treatments were able to retard aflatoxin formation in the fish tissues, to a safer level of lower than 4 µg/kg. This study’s findings have revealed the sustainability aspects, predominantly the conversion of agricultural products into value-added bio-additives in food processing and preservation.

## 1. Introduction

Fish is increasingly recognized as a significant source of natural protein because it contains lower cholesterol levels compared to other animal proteins and also provides appreciable amounts of bioactive compounds. Fish and fishery products contribute approximately 19% of protein intake, and fundamental to providing various micronutrients, particularly essential fatty acids [1]. Fish high level of nutraceutical properties compounds makes it a valuable part of a nutritionally adequate diet [2,3]. Omega-3 fatty acids, astaxanthin, and bioactive peptides, readily found in fish, are vital components that promote heart and brain health, safeguard human cells against oxidative stress, and reduce inflammation [4,5]. Marques et al. [6] and Iqra Nazir et al. [7] noted that, the primary therapeutic benefits of fish products are linked to their rich long-chain omega-3 fatty acids, vitamins, and minerals. Particularly, the Food and Agriculture Organization [FAO] stated that, global fish production stands at approximately 223 million tons, with about 163 million tons being consumed directly by people; while the rest is utilized for non-food purposes [FAO, 2024].

Scholars have proved that pathogenic activities are critical risks to food safety and storability, and these pathogenic microorganisms include bacteria, fungi, and viruses, which cause foodborne diseases. Foodborne microorganisms have been linked to mild to severe illnesses such as diarrhea, gastrointestinal spasm, cancer, miscarriage, and vomiting [1,8,9,10,11]. For instance, *Aspergillus* and *Penicillium*, which are found in some dried fish tissues, are known to be causative agents for liver cancer and suppressed immunity [12,13]. Additionally, *Staphylococcus aureus,* another bacterial strain commonly associated with dried fish, is a major cause of pneumonia, septicemia, impetigo, and diarrhea [14]. Aflatoxins (AFs), which are produced by some fungal strains (mainly *Aspergillus flavus* and *Aspergillus parasiticus*), are major challenges to the public health due to their carcinogenic effects [15,16,17]. Microbial activities and formation of these toxic substances in the fish body, is enhanced by hygroscopic nature of dried fish muscles, poor processing and storage conditions—warm and humid environments [18,Alameri *et al.*, 2023,Uguru *et al.*, 2023]. Aflatoxins have a lot of public health challenges, causing serious ailments such as, respiratory disorder, liver failure, hepatocellular carcinoma, and cancer [19,20,21,22]. Dried fish muscle as hygroscopic characteristics, leading to the hygroscopic uptake of moisture, which creates favorable conditions for microbial activity [12].

Fish products are susceptible to rapid deterioration in nutritional quality and microbial invasion, even when dried and stored in ambient temperature conditions. This vulnerability can be linked to pathogenic contamination, enzymatic reactions, lipid oxidation, and bio-antioxidants breakdown. These factors have adverse effects on public health, and economic issues [23,24]. Food preservation contributes substantially to nutritional security, as it inhibit any biochemical reactions, enhances food items shelf life, thereby maintaining a steady food supply throughout both the growing and off seasons [25]. Drying one of the most widely used fish preservation techniques, primarily due to its simplicity, minimal energy requirements, and accessibility [Uguru *et al.,* 2023]. Despite the numerous benefits of drying, challenges do arise. Nutrient loss over extended storage periods and vulnerability to cross-contamination from environmental pollutants are common [26,27]. *Staphylococcus aureus, Salmonella spp, Bacillus spp*., and *Listeria monocytogenes*, are some of common bacterial strains that are associated with preserved fish products, which can results in foodborne illnesses [8].

Bio-fortification food processing approach, is one of the strategies that is used to enhance fish products nutritional quality and storability of. This technique involves the utilization of essential oils (EOs) and plant extracts, as preservative and nutrients enhancers during fish processing operation [28,29,Rathod *et al.* 2021]. Also, the mechanistic effects of these bio-preservative, can be attributed to the large amount of phytochemical compounds they contain [30,24]. There are many environmental and food safety issues, that have been confirmed to be associated with artificial preservatives. Some of these health issues linked to synthetic preservatives include cancer, diabetics, allergenic reactions, respiratory problems, and heart problems [31,32,33,34,35]. Therefore, there is significant quest for rapid clinical investigations into safer and environmentally friendly preservatives, mainly of plant origin [36,37,38,39,29,40,41,42].

Most previous studies were based on the application of a single plant derivative formula, with little focus on the synergistic effects of hybridized multi-plant derivatives. Particularly, there is a dearth of information regarding the use of a single experimental framework for the simultaneous evaluation of different factors, such as nutritional enhancement and retention, antimicrobial effects, and inhibition of aflatoxin production. Therefore, this study is designed to achieve multiple interrelated objectives, based on the hybridization approach of different plant extracts and essential oils (EOs), which are to: (i) quantify the phytochemicals constituents of basic treatments (plant extracts and EOs), using validated spectrophotometric approach; (ii) determine the potential of treatments phytochemicals, in preserving dried fish nutrients during prolonged storage under ambient environmental conditions; (iii) evaluate the treatments antimicrobial effectiveness, mainly against microorganisms that can lead to microbial spoilage; (iv) determine the anti-aflatoxigenic efficacy of the treatments using validated HPLC approach; and (iv) examine the complementary potential of combined phytoconstituents. The vitamins retention analysis was incorporated into the experimental framework, to validate the antioxidant effects and mechanistic performance of the treatments, as ambient conditions facilitates nutrients’ oxidation, mainly fat-soluble vitamins. These approaches not only aim, to enhance the preservation and nutritional quality of fish, but also promote the sustainable use of agricultural waste products, as valuable resources for essential oils and other beneficial compounds. This differentiates this research from existing well-documented previous findings that mainly used the edible part of agricultural products.

## 2. Materials and methods

### 2.1. Materials

#### 2.1.1. Plant and fish materials.

The ginger (*Zingiber officinale*), banana (*Musa acuminate*), and turmeric (*Curcuma longa*) peels were sourced from a local market in Taif City. The matured catfish were procured from Wadi Mitna. The fish were washed with distilled water, placed in ice-cooled boxes and taken immediately to the laboratory. Also the lime (*Citrus aurantiifolia)* fruits were purchased from an orchard in Taif City, Saudi Arabia.

#### 2.1.2. Chemical and equipment.

The methanol, Selenite Cystine (SC) broth, Rappaport–Vassiliadis (RV) broth, Xylose Lysine Deoxycholate (XLD) agar, nutrient agar (NA), Baird-Parker agar (BPA), Potato Dextrose Agar (PDA), Mannitol Egg Yolk Polymyxin (MYP) agar, Aspergillus flavus/parasiticus agar (AFPA), Brilliant Green Agar (BGA) plates, and the ICP–OES system (model: iCAP PRO XPS) was produced by Thermo Fisher Scientific Inc. America. The HPLC system, model LC-W100B, was manufactured by Wincom Co. Ltd., China. Phosphate-buffered saline (PBS), and bromocresol green (BCG) were from Acros Organics (Waltham, MA, USA). Sodium carbonate (Na₂CO₃) was obtained from Merck KGaA, Darmstadt, Germany.

### 2.2 Methods

#### 2.2.1 Plant extract and oil preparation.

The collected raw materials were carefully sorted to remove any foreign matter, then oven-dried at 60°C. After drying, they were ground using a laboratory grinder and filtered with a 1.00 mm nylon filter. Then, extracts were prepared in compliance with established standards, using food-grade ethanol (98% purity) as the carrier solvent. The plant material powder was totally immersed in the ethanol at 28 ± 5°C for three days, and the mixture was agitated every six hours for ten minutes. After the three days, the mixture was sieved with a Grade 1 filter paper, and the solution obtained was concentrated in a water bath at 40°C, to evaporate the solvent (ethanol) to obtain a thick crude extract [43]. The food grade ethanol (98% purity) was selected over methanol, because of serious health challenges linked to methanol consumption [44].

Additionally, the essential oils were extracted using the steam distillation approach. This is to preserve the oil’s essential healthful qualities, and avoid the occurrence of chemical residues from the extracting solvents. About 3 kg of pulverized plant material was fed into a distillation chamber, and subjected to steam flow to vaporize the oil component, from the parent material. The vaporized oil was condensed, separated from the water with a separating funnel and dried at 45 ± 2^o^C for 20 minutes [45].

#### 2.2.2. Experimental treatment plans.

The concentrations of extracts and oils used in this study were chosen according to the range previously utilized by other researchers for essential oils and extracts [39,37]. The lime extract (LE) was integrated into some of the treatments, as a cross-check to establish if the LE can significantly enhanced the antimicrobial effects of the other treatments. Bio-additives with higher acidity level create favorable conditions, for essential oils and extracts to inhibit pathogenic activity, by increasing the acidity of the treatment medium [46]. Tap water was the solvent composition (carrier medium) for each treatment. The experimental design mix ratio is presented below:

Control:100% tap water (untreated borehole water)

T1:2% (w/w) of Lime extract (LE)

T2:2% (w/w) of ginger peel extract (GPE)

T3:2% (w/w) of turmeric peel extract (TPE)

T4:2% (w/w) of banana peel extract (PPE)

T5:1% (w/w) of ginger peel oil (GPO)

T6:1% (w/w) of turmeric peel oil (TPO)

T7:1% (w/w) of banana peel oil (PPO)

T8:2% GPE + 1% GPO + 2% LE

T9:2% TPE + 1% TPO + 2% LE

T10:2% PPE + 1% PPO + 2% LE

T11:1% GPO + 1% TPO + 2% LE

T12:1% GPO + 1% PPO + 2% LE

T13:1% TPO + 1% PPO + 2% LE

T14:2% GPE + 1% TPE

T15:2% TPE + 1% PPE

T16:2%GPE + 1% PPE

Where; LE – lime extract, GPE – ginger peel extract, GPO – ginger peel oil, PPE – banana peel extract, PPO – banana peel oil, TPE – turmeric peel extract, TPO – turmeric peel oil, T – treatment

#### 2.2.3. Fish samples preparation and storage.

The fish was skinned in the laboratory and the flesh recovered, which was cut into the size of about 100 g. The dripping method was used to apply the treatments to the fish samples, at a temperature of 15 ± 3^o^C. Each sample was immersed in the designated treatment dose for 4 hours, and then allowed to drip for 20 minutes before being dried in a laboratory oven (model: 637G, Fisher Scientific Inc., USA) at 115°C to attained a moisture level of about 8%.

The 16 fish specimens (both control and the treated) were kept in a container (31 ± 6^o^C, 87 ± 6% RH) for eight weeks (the experimental duration. To protect the fish from direct insect and pest exposure while allowing air circulation between the fish samples and the surrounding air, each container was covered with nylon mesh (0.3 µm).

#### 2.2.4. Quality assurance (QA) and quality control (QC).

The blank samples were mixed with the certified reference materials (CRMs) to achieve quality control. Similarly, aseptic and replicate plating methods were adopted, and the tests were performed in three replicates.

### 2.3. Laboratory examinations

#### 2.3.1. Moisture content (MC) evaluation.

The hydration status of the samples was determined through the gravimetric technique, in accordance with the Association of Official Analytical Chemists (AOAC) guidelines [AOAC, 2000]. Individual specimen was oven-dried by using the laboratory oven (model: 637G, Fisher Scientific Inc., USA), at a temperature of 100 ± 2°C until a constant mass was achieved. The moisture level in the fish tissues was computed by using Equation 1.


$$\text{Moisture content }(\text{MC})\%=\frac{{\text{W}}_{\text{2}}-{\text{W}}_{\text{3}}}{{\text{W}}_{\text{3}}-{\text{W}}_{\text{1}}}\times \text{100}(\text{wet basis})\;$$ (1)

Where:

W_1_ = Weight of moisture can with the lid (g),

W_2_ = Weight of wet fish sample + moisture can with the lid (g), and

W_3_ = Weight of dry fish sample + moisture can with the lid (g)

#### 2.3.2. Plant derivative pH determination.

The acidity (pH) level of the extracts and essential oils was measured in accordance with AOAC procedures, by employing a calibrated digital pH meter (model: pHCal, produced by Analab Scientific Instruments Pvt. Ltd., India). Particularly, the device was calibrated with these buffered media at pH 4.0, 7.0, and 9.0, before taking new measurement. After calibration, the electrode was thoroughly cleaned using distilled water, dried, and dipped into the specimen solution. Then, the pH value of the sample was read from the pH meter screen, after the reading stabilized.

#### 2.3.3. Minerals determination.

The calcium (Ca), phosphorus (P), iron (Fe), potassium (K), and zinc (Zn) concentrations in fish muscles (tissues), were determined in accordance with the AOAC 2020 official guidelines, by using the Inductively Coupled Plasma–Optical Emission Spectrometry (ICP–OES) approach. Specifically, these wavelengths were used for mineral determination: Ca – 422.7 nm, P – 213.6 nm, Fe – 259.9 nm, K – 766.5 nm, and Zn – 213.9 nm.

#### 2.3.4. Vitamins determination.

The carotenoids, vitamin A, total B vitamins, and vitamin E profiles in the EOs, extracts and fish samples were determined using an HPLC system. The fat-soluble vitamins (vitamins A and E) were quantified using the following HPLC parameters: using a C18 column with a column temperature of 31°C, a flow rate of 1.0 mL/min, an injection volume of 10 µL, a run time of 20 min, and UV detection at 325 nm for retinol, 450 nm for β-carotene, and 292 nm for α-tocopherol. Likewise, the water soluble vitamin (vitamin B) was measured using the following HPLC parameters: using a column temperature of 30°C, 0.8 mL/min flow rate, 20 µL injection volume, a run time of 25 min, and UV detection which ranged from 260 nm to 360 nm. The correlation coefficient (R²) value for vitamin A was 0.997, β-carotene was 0.998, α-tocopherol was 0.997, and B-vitamins were 0.995. The LOD and LOQ values for vitamin A were 0.02 and 0.20 µg/mL, β-carotene were 0.05 and 0.25 µg/mL, α-tocopherol was 0.02 and 0.10 µg/mL, and B vitamins ranged from 0.01 to 0.20 µg/mL. The relative standard deviation of less than 4%, and CRM recovery ranging from 93% to 105%.

#### 2.3.5. Protein determination.

The protein level in the dried fish specimens, was determined in accordance with AOAC (2000) standard, by employing the Kjeldahl flasks (produced by Fisher Scientific Inc. USA), and following the explanations provided by Al Banna et al. [27].

### 2.4. Phytochemical properties measurement

#### 2.4.1. Total saponin content (TSC).

The TSC values of the extracts, EOs, and water were measured using a UV–Vis spectrophotometer through the spectrophotometric approach. 1 mL of each sample was added to 1 mL of C_8_H_8_O_3_ (8% w/v) and 10 mL of 72% H_2_SO_4_. The product was placed in a water bath pre-set at 65 ± 1°C for 15 minutes, before the beaker was placed in an icy environment to quench the reaction. Then, a UV-Vis spectrophotometer was used to measure the product optical density at 540 nm, and the TSC was quantified as diosgenin mg DE/g, with diosgenin as the reference standard. These were the spectrophotometric analytical validation parameters: R^2^ of 0.9881, LOQ = 1.82 µg/mL, and LOD = 0.60 µg/mL, calibration range of 0–100 µg/mL, and RSD of <4% [47].

#### 2.4.2. Total curcuminoids content (TCC).

The TCC of the samples was determined through the spectrophotometric approach, with the aid of a UV–Vis spectrophotometer. 1 mL of the sample (water, extract, or EO) was mixed with 25 mL of ethanol, filtered through Grade 1 filter paper, and the optical density was measured at 425 nm, using ethanol as the blank. The spectrophotometric assay validation criteria were: R^2^ of 0.9891, LOD = 0.30 µg/mL, LOQ = 0.91 µg/mL. concentration range = 0–100 µg/mL, and RSD of <4%. The TCC was quantified as mg CE/g, where CE is the curcumin equivalent, using the curcumin solution as the reference standard.

#### 2.4.3. Total alkaloid content (TAC).

The TCC value was determined using a UV–Vis spectrophotometer, through the spectrophotometric method. 1 mL of the sample was dispersed in 10 mL of 2% ethanoic acid, sieved through Whatman No. 1 filter paper into a beaker containing 10 mL of BCG and 10 mL of PBS (pH 4.7), to produce an alkaloid–dye conjugate, which was later separated into the chloroform phase. The optical absorbance was recorded at 480 nm. Specifically, atropine was the standard reference, and these validation parameters were used: R² = 0.9872, LOQ = 1.52 µg/mL, and LOD = 0.50 µg/mL, analytical range of 0–100 µg/mL, and RSD of <4%. The TCC was expressed as mg AE/g, where AE is the atropine equivalent [48].

#### 2.4.4. Total cyanogenic glycosides (TCG).

The TGC was determined through the spectrophotometric technique. 1 mL of the sample was added to 5 mL of PBS (pH 6.5), and the solution was poured into a beaker containing nitrocellulose paper. The beaker (with its contents) was then placed inside a water bath at 34 ± 2°C for 10 hours to ensure complete reaction. The absorbance of the resulting solution was measured at 530 nm, using KCN as the reference. These validation parameters were used: R² = 0.9915, LOQ = 0.91 µg/mL, LOD = 0.30 µg/mL, and RSD less than 4%. The TGC was expressed as mg KCE/g, where AE is the atropine equivalent.

#### 2.4.5 Total stilbenoid level (TSL).

The TSL of the samples was measured using the spectrophotometric technique. 11 mL of the solution were prepared by dissolving 1 mL of the sample in 10 mL of methanol, which was closely followed by filtration. Then, the optical density of the filtrate was measured at 310 nm using a UV–Vis spectrophotometer. Trans-resveratrol was the reference, while these were the spectrophotometric analytical validation parameters: R^2^ was 0.9895, LOD was 0.25 µg/mL, LOQ was 0.80 µg/mL, and RSD less than 4%. The result unit was mg RE/g, where RE is resveratrol equivalent.

#### 2.4.6. Total phenolic acids (TPC).

The spectrophotometric technique was employed to measure the total phenolic content (TPC) of the samples. The sample was poured into a volumetric cylinder, and the volume was adjusted to 11 mL with methanol. The solution was sifted, and a 1 mL portion of it was added to 5 mL of 10% phenol reagent and 2 mL of 7.5% Na₂CO₃ solution. Thereafter, the product was kept at 25 ± 2^o^C for 1 hour, before the absorbance was taken at 750 nm, and the unit of the result was mg GAE/g [49]. These were the spectrophotometric analytical validation parameters: R^2^ of 0.9931, LOQ = 1.36 µg/mL, and LOD = 0.45 µg/mL, calibration range of 0–100 µg/mL, and RSD less than 3%

### 2.5. Microbial analysis

Specifically, there was no controlled inoculation procedure, before the fish samples were stored in this study. All the microorganisms’ loads investigated in this research were of natural origin, primarily resulting from anthropogenic actions such as handling and storage operations. Especially, all bacterial isolations were based on genus-level identification; hence, their identification was generally expressed at the species level (spp.).

#### 2.5.1. Total viable Bacterial Count (TVBC).

The Plate Count Agar (PCA) was used to determine the total viable bacteria load of the fish samples.

#### 2.5.2. Bacterial strains determination.

*Staphylococcus spp.*

Exactly 10 g of ground fish muscle was mixed into 90 mL of sterile buffered peptone water to produce a 1:10 dilution, which was later diluted to 10^−6^ using sterile diluent. 0.1 mL of the aliquot was dispersed onto the BPA plate, which was then cultured at 37°C for 24 h. The *Staphylococcus spp.* clusters were detected by their black to gray, shiny coloring, and their counts were recorded. The *Staphylococcus spp.* count was then calculated using Equation 1, and the results given as CFU/g.


$$population\;=\frac{\text{colony coun}\times \text{dilution factor}}{\text{plated weight}}\text{1}$$

*Salmonella spp.*

The aliquot was injected into SC (Selenite Cystine) broth and incubated at 40°C for 24 hours. 0.1 mL of the enriched broth was inoculated on an Xylose Lysine Deoxycholate Agar plate, and cultured at 40°C for 24 hours, and the *Salmonella spp*. colonies were recognized by their red coloration and counted.

*Bacillus spp.*

Exactly 0.1 mL of the aliquot was spread onto the Mannitol Egg Yolk Polymyxin Agar plates, which were incubated at 34°C for 24 hours. The *Bacillus spp.* was identified and enumerated based on its pink coloration, and the population was calculated using Equation 1.

*Listeria spp.*

The aliquot (0.1 mL) was spread over an Oxford agar plate, and maintained at 35°C for 48 hours. The clusters produced were identified by their grey-green coloration, and the population was counted and calculated using Equation 1.

#### 2.5.3. Fungal count.

The fish muscle (10 g) was mixed with sterile Buffered Peptone Water (90 mL) to produce a 1:10 dilution medium, which was later diluted to 10^−4^ using sterilized buffer. To determine the fungal count on the fish body, dilutions of the homogenized sample were plated on Potato Dextrose Agar (PDA) and incubated at 30°C for 4 days. Then, the colonies formed were counted using a digital colony counter (model J-3, manufactured in China), and the results were expressed as CFU/g.

#### 2.5.4. Fungal strains determination.

*Aspergillus spp. (black mold).*

The aliquot (0.1 mL) was streaked on PDA plate, and incubated at 28°C for 4 days, Then, the *black mold* was identified based on its globose conidial heads and the appearance of biseriate phialides.

*Aspergillus spp. (flavus group).*

Exactly 0.1 mL of the portion was spread on an AFPA plate and incubated at 27°C for 6 days, and the *Aspergillus spp. (flavus group),* growth was recognized by its green-to-olive coloration with rough conidial heads. The *Aspergillus spp. (flavus group),* fungal burden was computed by using the expression presented in Equation 1.

*Rhizopus spp.*

Precisely, 0.1 mL of the prepared sample was spread on a PDA plate and incubated at 28°C for 5 days. The *Rhizopus spp*. was then identified based on its cottony white mycelia. The *Rhizopus spp.* colonization levelwas calculated using Equation 1.

*Penicillum spp.*

The portion (0.1 mL) was surface-plated onto PDA plates and incubated in an oxygen-rich environment at 25°C for 8 days. The *Penicillium spp*. was then identified based on its powdery texture with a pale reverse.

#### 2.5.5. Aflatoxins determination.

The HPLC technique was used to detect and measure the population of the AFs in the dried fish samples in accordance with procedures described by Coskun [50], Water and 0.1% formic acid was the mobile phase A, while methanol and acetonitrile mixed at a ratio of 6:4 was the mobile phase B. Particularly, 28^o^C column temperature, 20 µL injection volume, 20 minutes run time, and 1.0 mL/min flow rate were some of the basic HPLC operating parameters, used to measure the AFB_1_ and AFB_2_ content in the fish tissues. Essential parameters used for the validation of the mycotoxin analysis are presented in Table 1, and the All AFB_1_ and AFB_2_ concentrations were expressed as µg/kg.

**Table 1:** Validation data.

Factor	AFB_1_	AFB_2_
**Linear range [µg/kg]**	0.1–20	0.1–20
**Coefficient of determination (R**^**2**^)	0.998	0.997
**LOD (limit of detection) [µg/kg]**	0.04	0.05
**LOQ (limit of quantification) [µg/kg]**	0.20	0.25
**Recovery rate [%]**	93–105	95–106
**RSD (Relative Standard Deviation) [%]**	< 4%	< 4%

### 2.6. Data analysis

The SPSS statistical software (IBM version 22.0) was utilized to scrutinize the raw laboratory results. The multivariate analysis of variance (ANOVA), was employed to establish the influence of the treatments, on the dietetic properties and microbial contamination levels of the fish sample. Basically, Shapiro–Wilk test was used to evaluate the normality. Thereafter, the Duncans’ multiple range test (DMRT), was used to separate the mean at 5% significant level (p < 0.05), due to this post-hoc sensitive in means separation of large volume of data that were obtained in this study. All laboratory tests were performed in triplicate (n = 3). Additionally, descriptive statistics, and dendrogram were further used to discuss the results.

## 3. Results and discussion

### 3.1. Chemical composition of the treatments

#### 3.1.1. Bioactive compounds composition.

Table 2 presents the bioactive compounds of the tap water (borehole water), plant oils and extracts used in fish preservation. These essential phytochemicals provide insight into the antimicrobial and preservative effects of the pant derivatives. It was observed that turmeric extract, contain the highest concentrations of curcuminoids, whereas banana peel extract and oil have the highest levels of saponins. Additionally, ginger extract showed the greatest amounts of alkaloids. Interestingly, cyanogenic glycoside was absent in both turmeric extract and oil, although trace amounts were found in the extracts and oils of banana and ginger. Notably, the tap water recorded no detectable phytochemical compounds.

**Table 2:** Results of tap water and essential oils and extract phytochemical screening.

Parameter	LE	GPE	TPE	PPE	GPO	TPO	PPO	Tap water
**TSC (mg DE/g)**	2.97 ± 0.45	4.37 ± 0.35	6.43 ± 0.67	14.23 ± 0.93	0.83 ± 0.15	1.43 ± 0.15	2.47 ± 0.45	ND
**TCC (mg DE/g)**	ND	ND	57.33 ± 3.51	ND	ND	22.67 ± 2.52	ND	ND
**TAC (**mg AE/g)	0.90 ± 0.20	5.23 ± 0.25	3.47 ± 0.25	4.67 ± 0.25	0.43 ± 0.15	0.67 ± 0.15	0.57 ± 0.06	ND
**TCG (mg KCE/g)**	ND	0.33 ± 0.15	ND	2.82 ± 0.42	ND	ND	0.47 ± 0.15	ND
**TSL (**mg RE/g)	ND	1.93 ± 0.42	2.40 ± 0.40	3.30 ± 0.20	ND	ND	ND	ND
**TPC (mg GAE/g)**	4.77 ± 0.38	22.07 ± 0.70	33.43 ± 0.81	42.53 ± 1.42	3.77 ± 0.42	8.83 ± 0.51	2.70 ± 0.36	ND

±- mean and the standard derivation; Total stilbenoid level (TSL); Total saponin content (TSC); Total curcuminoids content (TCC); Total alkaloid content (TAC); Total cyanogenic glycosides (TCG); Total phenolic acids (TPC); ND- Not detected

Curcuminoids possess strong antioxidant and antimicrobial properties, exhibiting broad-spectrum antimicrobial activity against numerous varieties of bacteria, fungi, and viruses, by causing disruption to the microbial cell membranes [51]. Curcuminoids have the capability of causing microbial membrane fluidity, resulting in the seepage of proteins and nucleic acids from the cells, thereby exposing the cells to redox imbalance, destabilization, and eventually death of the pathogen [52]. In addition, saponins and alkaloids, have shown potent antimicrobial efficacy against broad spectra of pathogens [53,54]. Saponins tend to inhibit biofilm formation, thereby depriving bacteria of vital nutrients and anchorage sources. Saponins have a hydrophilic chain that can attack the proteins and lipids constituents of the biofilm, thus increasing cell permeability and reducing the cohesiveness of the biofilm. Alkaloids are capable of obstructing enzymatic actions and receptor molecules, as well as depriving microorganisms of essential amino acids. This hinders the cells’ survival [55]. These mechanistic actions will collectively result in significant decline microbial activities in the preserved fish samples, during the prolonged storage duration. This analysis of these chemical composition profiles indicated that, turmeric products exhibit the strongest antimicrobial properties, while having the least anti-nutritional factors.

#### 3.1.2. Vitamins and pH composition.

Table 3 displays the compositions of pH, carotenoids, vitamins B and E, in the plant oils and extracts used for fish preservation. It was observed that TPE and PPE generally have the higheer carotenoids concentrations; PPE products recorded the maximum B vitamin level, while GPO had the highest vitamin E concentration. These vitamins contribute not only to the nutritive enhancement of the preserved fish but also offer additional preservation benefits due to their antioxidant properties. Vitamins B complex and E support microbial growth inhibition, indirectly contributing to preservation by minimizing spoilage [56]. Higher concentrations of B vitamins retard the survival of spoilage microorganisms, although this effect can be extremely reliant on the overall composition of the food matrix and the microbial community that is present [57]. Though vitamins A and E have the ability of enhancing microbial metabolism, these vitamins safeguard against oxidative degradation, thus prolonging the fish shelf life and maintaining their quality [58]. Particularly, these vitamins will enrich the vitamin and antioxidant content of the processed fish samples, through diffusion into fish tissues, surface binding with the fish muscles, and synergistic stabilization of the fish bioactive compounds. These actions will help to avert the fish fat rancidity, hence, preserving the fish integrity during storage [3,59].

**Table 3:** Essential oils and extract vitamins profile.

Parameter	Carotenoids (mg/100g)	Vitamin B (mg/100g)	Vitamin E (mg/100g)	pH
**LE**	2.62^a^ ± 0.04	4.31^c^ ± 0.03	2.41^a^ ± 0.03	3.23^a^ ± 0.09
**GPE**	3.74^b^ ± 0.09	5.02^de^ ± 0.03	3.21^b^ ± 0.02	5.25^b^ ± 0.19
**GPO**	2.59^a^ ± 0.02	2.32^a^ ± 0.01	47.12^e^ ± 1.06	5.92^c^ ± 0.22
**PPE**	11.15^f^ ± 0.07	5.84^e^ ± 0.08	3.67^bc^ ± 0.03	6.28^d^ ± 0.11
**PPO**	4.63 cd ± 0.03	2.81^b^ ± 0.04	40.25^d^ ± 0.11	6.64^e^ ± 0.17
**TPE**	18.93^f^ ± 0.17	4.65^c^ ± 0.11	4.03^c^ ± 0.04	5.17^b^ ± 0.19
**TPO**	5.27^d^ ± 0.04	2.43^ab^ ± 0.04	77.39^f^ ± 2.35	5.96^c^ ± 0.14

Mean ±standard deviation (n = 3); values having similar alphabet in the same column (nutrient) signifies that the means are not significant different (p ≤ 0.05) according to DMRT

### 3.2. Nutritional quality of the fresh fish flesh

Table 4 presents the concentrations of vitamins, proteins, and essential minerals in the fish flesh used in this study. Typically, this nutritional level of the unprocessed fish was elevated to serve as the baseline reference, in order to assess whether the treatments have a substantial impact on enhancing the nutritional level of the processed fish samples. The fresh fish specimen contains significantly higher vitamin A, compared to vitamins B, D, and E; protein concentration of 44.67%; as well as Ca, P, Fe, K, and Zn concentration of 3416.00, 4042.33, 349.33, 2113.67, and 4.20 mg/kg, respectively. These results support the previous findings of Bogard et al. [60], for various fish species. The protein level recorded in this study was substantially greater, than the 13.59% value reported by Aberoumand et al. [61] for fresh *Oncorhynchus mykiss*. Essential nutrients such as protein, vitamins, and minerals are critical dietary indicators for good health [62]. The differences in the fresh fish nutritional level observed in this study, compared to the results documented by previous scholars, can be attributed to factors such as fish genotype, age, habitat, environmental pollution, and climatic conditions.

**Table 4:** The fresh fish muscle nutritional quality.

Parameter	Concentration
Vitamin A (mg/kg)	38.35^c^ ± 8.92
Vitamin B (mg/kg)	0.16^a^ ± 0.03
Vitamin D (mg/kg)	0.39^a^ ± 0.043
Vitamin E (mg/kg)	2.11^b^ ± 0.07
Proteins (%)	44.67^d^ ± 7.51
Ca(mg/kg)	3416.00^g^ ± 95.59
P (mg/kg)	4042.33^h^ ± 66.61
Fe (mg/kg)	349.33^e^ ± 11.15
K (mg/kg)	2113.67^f^ ± 79.53
Zn (mg/kg)	4.20^c^ ± 0.04

Mean ±standard deviation, replication – 3; means having similar alphabet within the same column implies that significant difference does not exist among the means (p ≤ 0.05), according to DMRT

### 3.3. The fish nutritional quality after storage

**Fig 1:**
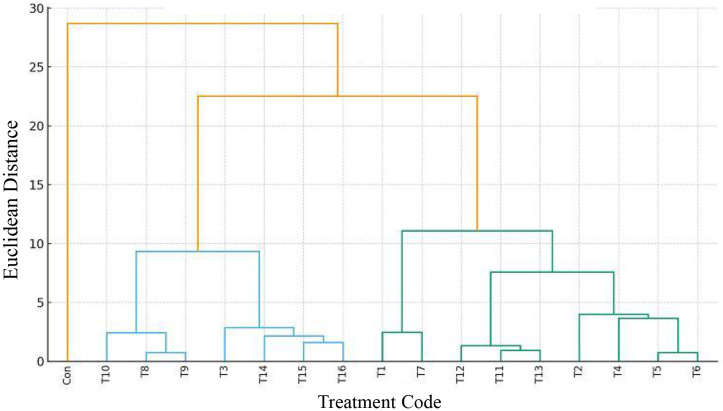
The Dendrogram of the fish samples nutritional depreciation rate during storage (week 8).

Table 5 and Fig 1 present the results of the dietary compositions of the processed and preserved fish flesh. It was noted that treatment and drying operations have a substantial effect, on the nutritional quality of the fish flesh, when Table 5 is compared with Table 4. The presence of essential bioactive compounds such as carotenoids, vitamins, saponins, curcuminoids, alkaloids, stilbenoids, phenolic acids, in the treatments, may increase the vitamins content in fish tissues, during the sample processing operation (steaming and drying). The higher vitamins concentration in the treatments (Table 3), can directly increase the vitamins level, in the fish samples through the diffusion process. Also, the infusion of basic phytochemicals, into the fish tissues will enhanced nutrients retention, by inhibiting the thermal and oxidative decomposition of the nutrients, mostly during the heat processing and storage operations [41,63]. The protein content recorded in this research was far less than the value of 70.28% reported by Aberoumand and Ziaei-Nejad [64]. The vitamin level recorded in this study supports the findings of Peycheva et al. [65] for processed canned fish samples; while the protein content of the control unit aligned with the results obtained by Sroy et al. [66] for dried fish samples.

**Table 5:** The processed fish samples nutritional quality before and after experimental period.

Treat	Vitamin A (mg/kg)		Vitamin B (mg/100g)		Vitamin D (mg/kg)		Vitamin E (mg/kg)		Proteins (%)	
	Wk0	Wk8	Wk0	Wk8	Wk0	Wk8	Wk0	Wk8	Wk0	Wk8
**Con**	30.19^a^ ± 0.10	23.94^b^ ± 0.05	0.152^a^ ± 0.002	0.130^b^ ± 0.002	0.307^a^ ± 0.003	0.253^b^ ± 0.004	2.04^a^ ± 0.03	1.69^b^ ± 0.04	42.17^a^ ± 0.02	35.05^b^ ± 0.03
**T1**	31.22^a^ ± 0.09	27.11^b^ ± 0.05	0.161^a^ ± 0.002	0.141^b^ ± 0.002	0.315^a^ ± 0.003	0.273^b^ ± 0.004	2.19^a^ ± 0.03	1.95^b^ ± 0.04	42.19^a^ ± 0.02	36.98^b^ ± 0.03
**T2**	44.31^a^ ± 0.08	40.27^b^ ± 0.24	0.192^a^ ± 0.004	0.171^b^ ± 0.004	0.386^a^ ± 0.009	0.345^b^ ± 0.009	2.23^a^ ± 0.05	2.11^b^ ± 0.02	42.19^a^ ± 0.03	38.99^b^ ± 0.07
**T3**	49.27^a^ ± 0.22	45.73^b^ ± 0.09	0.211^a^ ± 0.003	0.196^b^ ± 0.002	0.427^a^ ± 0.009	0.386^b^ ± 0.004	2.25^a^ ± 0.03	2.10^b^ ± 0.05	42.21^a^ ± 0.05	39.12^b^ ± 0.03
**T4**	42.61^a^ ± 0.11	38.46^b^ ± 0.09	0.231^a^ ± 0.005	0.211^b^ ± 0.003	0.466^a^ ± 0.013	0.411^b^ ± 0.008	2.24^a^ ± 0.04	2.05^b^ ± 0.06	42.15^a^ ± 0.04	38.49^b^ ± 0.10
**T5**	42.22^a^ ± 0.09	37.84^b^ ± 0.04	0.172^a^ ± 0.002	0.153^b^ ± 0.004	0.346^a^ ± 0.002	0.311^b^ ± 0.011	2.17^a^ ± 0.02	2.01^b^ ± 0.04	42.18^a^ ± 0.05	38.01^b^ ± 0.08
**T6**	46.92^a^ ± 0.05	42.09^b^ ± 0.05	0.191^a^ ± 0.004	0.171^b^ ± 0.003	0.446^a^ ± 0.007	0.402^b^ ± 0.007	2.19^a^ ± 0.03	2.03^b^ ± 0.05	42.16^a^ ± 0.04	38.16^b^ ± 0.16
**T7**	40.70^a^ ± 0.08	35.91^b^ ± 0.04	0.220^a^ ± 0.004	0.194^b^ ± 0.003	0.508^a^ ± 0.010	0.443^b^ ± 0.011	2.23^a^ ± 0.02	2.01^b^ ± 0.03	42.15^a^ ± 0.02	37.59^b^ ± 0.03
**T8**	47.22^a^ ± 0.11	44.85^b^ ± 0.04	0.210^a^ ± 0.004	0.200^gh^ ± 0.030	0.426^a^ ± 0.008	0.403^b^ ± 0.062	2.21^a^ ± 0.06	2.11^b^ ± 0.03	42.18^a^ ± 0.04	39.85^b^ ± 0.05
**T9**	53.68^a^ ± 0.04	51.13^b^ ± 0.09	0.221^a^ ± 0.004	0.210^b^ ± 0.004	0.384^a^ ± 0.007	0.365^b^ ± 0.007	2.22^a^ ± 0.06	2.11^b^ ± 0.06	42.17^a^ ± 0.02	39.93^b^ ± 0.06
**T10**	45.93^a^ ± 0.06	43.14^b^ ± 0.02	0.251^a^ ± 0.005	0.241^b^ ± 0.004	0.444^a^ ± 0.010	0.423^b^ ± 0.005	2.25^a^ ± 0.03	2.17^b^ ± 0.03	42.19^a^ ± 0.03	39.56^b^ ± 0.06
**T11**	45.54^a^ ± 0.04	41.99^b^ ± 0.06	0.181^a^ ± 0.003	0.165^b^ ± 0.003	0.365^a^ ± 0.007	0.335^b^ ± 0.007	2.18^a^ ± 0.03	2.00^b^ ± 0.03	42.18^a^ ± 0.02	37.72^b^ ± 0.05
**T12**	47.08^a^ ± 0.03	43.13^b^ ± 0.05	0.190^a^ ± 0.004	0.172^b^ ± 0.003	0.384^a^ ± 0.010	0.352^b^ ± 0.006	2.19^a^ ± 0.05	1.99^b^ ± 0.03	42.13^a^ ± 0.06	37.65^b^ ± 0.04
**T13**	43.21^a^ ± 0.13	39.90^b^ ± 0.05	0.211^a^ ± 0.003	0.193^b^ ± 0.002	0.425^a^ ± 0.007	0.390^b^ ± 0.006	2.21^a^ ± 0.05	2.01^b^ ± 0.08	42.16^a^ ± 0.03	37.57^b^ ± 0.06
**T14**	48.55^a^ ± 0.04	45.03^b^ ± 0.07	0.210^a^ ± 0.004	0.197^b^ ± 0.002	0.423^a^ ± 0.006	0.392^b^ ± 0.005	2.21^a^ ± 0.06	2.08^b^ ± 0.03	42.16^a^ ± 0.04	39.09^b^ ± 0.04
**T15**	51.21^a^ ± 0.04	47.85^b^ ± 0.04	0.217^a^ ± 0.009	0.201^b^ ± 0.005	0.436^a^ ± 0.018	0.407^b^ ± 0.014	2.22^a^ ± 0.03	2.06^b^ ± 0.05	42.17^a^ ± 0.03	39.23^b^ ± 0.06
**T16**	49.59^a^ ± 0.07	45.91^b^ ± 0.07	0.24a ± 0.003	0.224^b^ ± 0.006	0.482^a^ ± 0.004	0.444^b^ ± 0.010	2.24^a^ ± 0.05	2.07^b^ ± 0.05	42.19d^a^ ± 0.04	39.14^b^ ± 0.07

Mean ±standard deviation (n = 3); means having similar alphabetical letters, in the same column, indicates that significant difference does not exist among the means (p ≤ 0.05) according to DMRT.

It was noted that there was measurable nutrient depletion, in the fish samples after storage; though the treatments played a critical role in preventing this nutrients degradation. The anti-nutrients degradation mechanistic actions were dependent on the treatment type and concentration. Fish specimen exclusively treated with LE (T1), experienced the maximum nutrients depreciation, with its vitamins A, B, D, E, and protein levels, declining by 13.16, 12.42, 13.33, 10.96, and 12.35%, respectively, after the 8 storage weeks. Conversely, the treatments containing higher extract concentrations (T8, T9, and T10) displayed the minimum nutrient depreciation during storage, affirming the positive impact of hybridized treatments, in preventing nutrient loss from the fish tissues. The three-way hybridization can significantly improve the synergistic interactions of the three additives, thus increasing the bioactive compound concentration and antioxidant potency. Also, this multiple bio-additives hybridization, promotes the biosynthesis of new potent phytochemicals (conjugated compounds), resulting in better nutrient stabilization mechanisms [39,67,68,69]. The higher nutrient degradation observed in the T11 to T13 samples, although they are three-way hybridization, can be linked to the nature (essential oils) and concentrations (1%) of the additives. Essential oils tend to contain lower amounts of antioxidant compounds, compared to plant extracts; therefore, extracts have greater antioxidant effects on nutrients degradation than the essential oils [38,30,70]. Interesting, this study’s findings have revealed that plant derivatives can be successfully used to inhibit nutrient depreciation, which is a major problem in fish storage, as reported by Aberoumand et al., [71].

Apart from the anti-oxidative effects of phytochemical compounds, these compounds have potent anti-microbial action. This helps prevent microbial colonization of the fish muscles during storage, which might lead to the deterioration (biodegradation) of essential nutrients, through the production of aldehydes, amines, and organic acids [Mei *et al.,* 2019,^8^,^72^]. According to the dendrogram (Fig 1), the Control unit and T1 displayed the lowest anti-nutrients degradation ability, while hybridized treatments (T2 to T16) displayed excellent anti-nutrients oxidation potentials. This depicted that the combined treatments were highly effective in retarding the nutrients oxidation during storage [73]. Treatments T1 and T7 have a cluster with the highest vitamin degradation values (worst preservation), while T5 and T6 cluster indicates relatively higher vitamin degradation rates, but exhibit better preservation potential than T1 and T7.

Lime, ginger peel, and banana peel extracts and oils are rich in phytochemicals, with potent antioxidant capacity [74]. These active ingredients can substantially enhances the fish’s antioxidant properties, by infusing essential bioactive compounds into the fish tissues during processing [75,76]. Phenolic acids, curcuminoids, and saponins are highly effective in protecting the fish tissues, against fluidity and lipid peroxidation, mainly through metal chelation and radical quenching. These mechanistic actions can minimize nutrients leakage, and oxidation of vitamins and proteins, within the fish muscles during storage [77,78,79,80]. According to Kumar et al. [10], high contents of phenolics, flavonoids, and terpenoids have the potential to retard oxidative reactions, thereby preserving fish nutrients during storage.

### 3.4. Microbiology of the stored fish samples

#### 3.4.1. Total viable bacteria.

The bacterial populations in the fish samples results are presented in Table 6. The findings revealed that the treatments played significant roles, in inhibiting bacteria loads in the fish tissues during storage (p ≤ 0.05). It was T8, T9, and T10 samples, consistently maintained the minimum viable bacterial populations during the storage (excellent antimicrobial effect). This affirms the antimicrobial potency of these treatments used in this research, as their viable bacterial populations were lower than 300 CFU/g at week 8. Also, the control unit recorded the maximum total viable bacterial count (TVBC), attaining 1094 CFU/g at the eight week. T1 and T7 enriched fish samples portrayed weaker anti-bacterial action. The elevated bacterial count in the control unit, affirms the vulnerability of unpreserved fish tissues to bacterial intrusion during storage, supporting previously published reports by Tayel et al. [24]. Unprocessed fish tissues are susceptible to rapid microbial enzymatic reactions, leading to tissue decay, thereby reducing shelf life and the nutritional quality of the fish product [81].

**Table 6:** The total viable bacteria count (x10^2^ CFU/g).

Treatment	Storage (weeks)				
	0	2	4	6	8
**Con**	0.00	1.45^i^ ± 0.07	2.46^j^ ± 0.16	5.70^j^ ± 0.12	10.94^i^ ± 0.10
**T1**	0.00	0.98^h^ ± 0.08	1.92^i^ ± 0.06	3.79^i^ ± 0.05	5.11^h^ ± 0.17
**T2**	0.00	0.50^f^ ± 0.03	1.41^de^ ± 0.02	2.89^f^ ± 0.06	3.83^f^ ± 0.11
**T3**	0.00	0.32^de^ ± 0.05	1.11^b^ ± 0.09	2.44^e^ ± 0.10	3.43^de^ ± 0.11
**T4**	0.00	0.58^f^ ± 0.11	1.52^f^ ± 0.07	3.33^h^ ± 0.08	4.12^f^ ± 0.08
**T5**	0.00	0.30c ± 0.07	1.46^e^ ± 0.12	2.55^e^ ± 0.07	3.22^c^ ± 0.12
**T6**	0.00	0.39e ± 0.04	1.51^f^ ± 0.18	2.34^d^ ± 0.08	3.74^f^ ± 0.07
**T7**	0.00	0.50^f^ ± 0.07	1.76^g^ ± 0.07	2.71^f^ ± 0.08	3.42^de^ ± 0.07
**T8**	0.00	0.22^b^ ± 0.06	1.17^b^ ± 0.04	1.65^b^ ± 0.12	2.74^b^ ± 0.19
**T9**	0.00	0.20^a^ ± 0.03	0.84^a^ ± 0.06	1.43^a^ ± 0.06	2.33^a^ ± 0.06
**T10**	0.00	0.28^c^ ± 0.05	1.18^b^ ± 0.06	1.82^b^ ± 0.05	2.87^bc^ ± 0.16
**T11**	0.00	0.58^f^ ± 0.12	1.43^e^ ± 0.08	2.67^f^ ± 0.08	3.53^e^ ± 0.11
**T12**	0.00	0.31 cd ± 0.05	1.44^e^ ± 0.09	2.20^de^ ± 0.16	3.26^d^ ± 0.06
**T13**	0.00	0.62^g^ ± 0.06	1.75^g^ ± 0.09	2.76^g^ ± 0.10	3.51^e^ ± 0.07
**T14**	0.00	0.41^g^ ± 0.04	1.49^e^ ± 0.06	1.89^c^ ± 0.09	3.76^f^ ± 0.69
**T15**	0.00	0.34^d^ ± 0.04	1.20^c^ ± 0.04	2.11^d^ ± 0.07	3.91^d^ ± 0.12
**T16**	0.00	0.33^d^ ± 0.04	1.21^c^ ± 0.04	2.31^de^ ± 0.06	4.12^g^ ± 0.03

Mean ±standard deviation (n = 3); in the same columns, means with the alphabet represent significant indifference (p ≤ 0.05) according to DMRT

This study’s results reflect that the three-ways hybridization of LE with the other plants extracts or EOs ((T8 to T13), enhanced these treatments antibacterial potency. This is because the lime extract’s acidity level provides a conducive environment, which enhances the anti-bacterial performance of the phytochemical compounds in the other bio-additives. Lime extract has a lower pH value due to its high citric acid and ascorbic acid content, and some bacterial strains, such as *Staphylococcus spp., Salmonella spp., Bacillus spp*., and *Listeria spp.*, are very sensitive to acidic media. The acidic environment tends to weaken the bacteria’s cell walls, disrupts the osmotic pressure, thereby impairing their function [82,83,84]. According to Nieto Marín et al. [85], acidic environment tends to increase microorganisms’ cell membrane permeability, hence enhancing the penetration (infiltration) rate of the essential bioactive compounds of the treatments, leading to improved antimicrobial effect and storability.

Remarkably, the smaller bacterial counts recorded in T3, T6, T9, T11, T13, T14, and T15, in fish samples at the eight week, can be linked to the significant concentrations of vitamins, phenolic acids, saponins, curcuminoids, and stilbenoids in the additives products used (Tables 2, 3). Curcuminoids are potent antibacterial compounds, with the ability to disrupt the bacterial membrane’s impermeability, thereby causing leakage of intracellular constituents, and resulting in cellular metabolic dysfunction and death of the bacterial cell [86]. Additionally, curcuminoids have a strong ability to chelate heavy metals, thereby distorting the microbial respiratory system, and reducing the availability of non-essential nutrients to the microorganism. Saponins generate free radicals (H_2_0_2_, OH.), which causes oxidative stress to the cells. Phenolic acids greatly impair amino acid and ATP production in bacterial cells, leading to a massive shortfall in energy and genomic metabolism, thereby inhibiting bacterial growth and activity [87]. Phenolic acids have strong ability to destroy bacterial cell membranes, thereby increasing membrane permeability and leakage of essential nutrients. Also, phenolic acids’ metal chelation and enzyme suppression, can affect the nutrients’ availability to the bacterial cells [88]. Saponins can cause impairment the cell’s DNA and protein structures, resulting in nucleotides degradation and lipid peroxidation, which can lead to cell rupture [89,86]. Active phytochemical compounds possess strong antimicrobial properties, hence retarding bacterial growth and enhancing food storability [90,53]. This creates protective barriers around the fish tissues, resulting in antimicrobial activity and preventing nutrient degradation [29,68,40,91]. Vitamins are antioxidants with the ability to mitigate oxidative stress, thereby enhancing fish nutritional integrity and creating a bacteriostatic environment. These mechanistic actions enhance high bacterial mortality rate, and this can be linked to the lower bacterial counts in the treated fish samples [92,75].

Generally, the microbial counts recorded in this study were lower than those reported by Birie et al. [93], Al Banna et al. [27], and Rana et al. [94] for dried fish samples; however, this study’s findings were similar to the observations stated by Majumdar et al. [95] and Jensen et al. [59]. The variations in the microbial load reported by the various authors can be attributed to storage conditions, type of treatment(s) applied, experimental errors, and the methodology used [96,97].

#### 3.4.2. Isolated bacterial strains.

Table 7 presents the results of bacteria isolated from the fish tissue, both prior to and following the storage period. It was noted that the treatments had a significant impact on the pathogenic growth (p ≤ 0.05). The use of plant extracts and oils as preservatives effectively inhibited the development of disease-causing agents, thereby enhancing the safety and quality of the preserved fish. The results revealed that the preserved fish samples, showed microbial populations within the following ranges: *Staphylococcus spp.* at 41–91 CFU/g, *Salmonella spp* at 14–29 CFU/g, *Bacillus spp.* at 3–14 CFU/g, and *Listeria spp.* at 5–19 CFU/g. These bacterial counts demonstrated that *Staphylococcus spp.* had the highest population, whereas *Bacillus spp.* had the lowest in the enriched fish samples. Consistent with the TVBC readings, the Control and T1 exhibited higher *Staphylococcus spp., Salmonella spp., Bacillus spp.*, and *Listeria spp.* populations during storage. This can be linked to the lower amounts of bioactive compounds in T1, and the absence of photochemical compounds in the borehole water (Table 2) used in the Control unit. Remarkably, the results of this study aligned with the documented findings of Aberoumand and Baesi [98], who noted that saltwater treatment disrupts microbial growth in preserved Kotr fish.

**Table 7:** Isolated bacteria in the stored fish (x10^2^ CFU/g).

Treatment	*Staphylococcus spp.*		*Salmonella spp*		*Bacillus spp.*		*Listeria spp.*	
	Wk 0	Wk 8	Wk 0	Wk 8	Wk 0	Wk 8	Wk 0	Wk 8
**Control**	0.00	1.27^g^ ± 0.06	0.00	0.40^f^ ± 0.44	0.00	0.22^a^ ± 0.01	0.00	0.32^e^ ± 0.02
**T1**	0.00	0.91^f^ ± 0.08	0.00	0.29^e^ ± 0.19	0.00	0.14^ab^ ± 0.01	0.00	0.19^d^ ± 0.05
**T2**	0.00	0.62^bcde^±0.10	0.00	0.22^d^ ± 0.24	0.00	0.08^b^ ± 0.02	0.00	0.13^bc^ ± 0.02
**T3**	0.00	0.60^bcde^±0.09	0.00	0.21^d^ ± 0.23	0.00	0.12^b^ ± 0.02	0.00	0.18^c^ ± 0.03
**T4**	0.00	0.61^bcde^±0.09	0.00	0.20 cd ± 0.23	0.00	0.06^b^ ± 0.02	0.00	0.10^ab^ ± 0.03
**T5**	0.00	0.41^a^ ± 0.04	0.00	0.14^a^ ± 0.15	0.00	0.04^b^ ± 0.01	0.00	0.10^ab^ ± 0.03
**T6**	0.00	0.58^bcd^ ± 0.07	0.00	0.16^ab^ ± 0.18	0.00	0.04^b^ ± 0.03	0.00	0.06^a^ ± 0.04
**T7**	0.00	0.69^de^ ± 0.09	0.00	0.16^ab^ ± 0.18	0.00	0.07^b^ ± 0.04	0.00	0.10^ab^ ± 0.06
**T8**	0.00	0.52^ab^ ± 0.03	0.00	0.14^a^ ± 0.16	0.00	0.03^b^ ± 0.01	0.00	0.05^a^ ± 0.03
**T9**	0.00	0.53^abc^ ± 0.06	0.00	0.15^ab^ ± 0.17	0.00	0.05^b^ ± 0.01	0.00	0.07^ab^ ± 0.02
**T10**	0.00	0.59^bcd^ ± 0.08	0.00	0.15^ab^ ± 0.16	0.00	0.07^b^ ± 0.03	0.00	0.08^ab^ ± 0.05
**T11**	0.00	0.53^abc^ ± 0.08	0.00	0.16^ab^ ± 0.18	0.00	0.06^b^ ± 0.02	0.00	0.09^ab^ ± 0.03
**T12**	0.00	0.60^bcde^±0.08	0.00	0.18^bc^ ± 0.20	0.00	0.08^b^ ± 0.04	0.00	0.10^ab^ ± 0.06
**T13**	0.00	0.67^cde^ ± 0.07	0.00	0.16^ab^ ± 0.17	0.00	0.07b ± 0.01	0.00	0.10^ab^ ± 0.02
**T14**	0.00	0.67^de^ ± 0.03	0.00	0.22^d^ ± 0.24	0.00	0.08^b^ ± 0.03	0.00	0.11^ab^ ± 0.04
**T15**	0.00	0.73^e^ ± 0.08	0.00	0.22^d^ ± 0.24	0.00	0.07^b^ ± 0.50	0.00	0.14^ab^ ± 0.04
**T16**	0.00	0.66^cde^ ± 0.08	0.00	0.21 cd ± 0.23	0.00	0.07^b^ ± 0.01	0.00	0.11^ab^ ± 0.02

Mean ±standard deviation (n – 3); in the same column, means with different common letter represent significant difference according to DMRT (p ≤ 0.05)

The optimized antibacterial performance observed mainly in the T8–T13 samples, can be attributed to the collaborative antibacterial mechanistic actions, between the hybridized bio-additives bioactive compounds. Curcuminoids compound is highly effective against Gram-positive bacteria, as it retards cellular partitioning, mitosis, and quorum sensing of *Staphylococcus spp.* Curcuminoids also destroy the *Staphylococcus spp.* Murein layer, resulting in the massive seepage of the cell nutrients, and thereby leading to cell death [86]. Also, Curcuminoids have a broad spectrum of action against Gram-negative bacteria, and they can inhibit the production of ATP and motility genes in *Salmonella spp*. These actions cause severe free radical damage to the cells, which can result in a high mortality rate of *Salmonella spp.* bacteria [99]. Additionally, phenolic acids have strong antimicrobial action against Gram-positive bacteria such as *Bacillus spp.*, as they severely inhibit protein production within the cell, interrupt spore production, and disrupt enzymatic reactions. This causes a deficiency of spores, and a lower rate of *Bacillus spp.* reproduction [100].

Apart from the low pH of LE, which corrodes the bacterial cell membrane, leading to increase permeability and excessive seepage of intracellular constituents, lemon extract contains substantial amounts of monoterpenes and phenolics, which can interfere with cellular energy metabolism and enzymatic actions. Monoterpenes have the potential to disrupt lipid, amino acid, and protein production, and also creating respiratory disorders, hence causing serious damage to bacterial cells [101]. Therefore, the hybridization of these mechanistic activities (enzymes suppression, metabolic blockade and cell wall destabilization), can enhance the antimicrobial effectiveness of other plant-derived antibacterial treatments [102].

Also, the lower nutrient levels recorded in the Control and T1 samples can partially contribute to the high invasion of *Staphylococcus spp., Salmonella spp., Bacillus spp.,* and *Listeria spp.* in the fish tissues. For instance, some nutrients such as vitamins have antibacterial effects, which act as defense mechanisms against pathogenic invasion [41]. Interestingly, this laboratory investigation results buttressed the previous findings of Kunová et al. [29], on the utilization of bio-additives in preventing fish spoilage. Notably, T5, T6, T8, and T9 exhibited greater potency for inhibiting *Salmonella spp*. and *Listeria spp.* growth to negligible levels; T6 demonstrated greater effectiveness against *Bacillus spp.* and *Listeria spp.*, while T4 displayed a strong anti- *Staphylococcus spp*. activity. This affirmed the synergistic antimicrobial effects of the plant extracts and plant oils, and potentiation action of the lime extract. This study has revealed that plant derivatives can be used as a potential substitute for synthetic antibacterial preservatives, leading to the enhancement of food safety and public health, as previously documented by Christaki et al. [38] and Petcu et al. [92].

#### 3.4.3. Fungal contamination.

**Fig 2:**
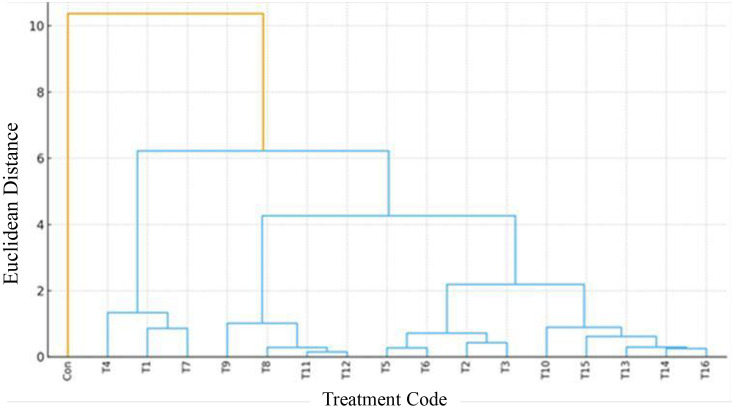
The dendrogram of the treatments and total fungal count.

The outcomes of the total fungal count are given in Table 8 and Fig 2, and the results indicated that the different treatment options significantly impacted fungal survival within the fish tissue. During the eight-week storage period, the fish samples in the control group exhibited the highest total fungal population, reaching a total viable fungal count (TFC) of 1176 CFU/g. Similarly, fish muscles fortified with bio-extracts and EOs (T1 to T16), demonstrated suppressed fungal reproduction; though each treatment exhibited different anti-fungal effectiveness. Furthermore, the dendrogram plot (Fig 2) validates the various treatments’ antifungal efficacy. T8, T9, T11, and T12 verified strong efficacy; T13, T14, T15, and T16 showed moderate efficacy; while T1, T4, and T7 exhibited the lowest antifungal efficacy. Above all, the control group displayed the lowest antifungal efficacy. The high fungal proliferation, associated with control experimental group, highlights the vulnerability of untreated fish muscles to fungal infection (2017). Similarly, Zhang et al. [103]

**Table 8:** The total fungal count (x10^2^ CFU/g).

Treatment	Storage duration (weeks)				
	0	2	4	6	8
**Con**	0.00	1.67^h^0.09	3.72^j^ ± 0.04	5.53^j^ ± 0.22	11.76^j^ ± 0.12
**T1**	0.00	0.81^g^ ± 0.07	2.93^i^ ± 0.08	4.08^i^ ± 0.08	7.37^i^ ± 0.06
**T2**	0.00	0.63^e^ ± 0.04	1.70^h^ ± 0.03	2.34^h^ ± 0.07	4.54^h^ ± 0.08
**T3**	0.00	0.66^d^ ± 0.02	1.91^g^ ± 0.12	2.63^g^ ± 0.05	4.76g ± 0.07
**T4**	0.00	1.18^f^ ± 0.07	2.59^i^ ± 0.08	3.18^i^ ± 0.08	6.27^i^ ± 0.06
**T5**	0.00	0.21^a^ ± 0.04	1.66^f^ ± 0.08	2.24^f^ ± 0.11	4.75^f^ ± 0.15
**T6**	0.00	0.19^a^ ± 0.05	1.73 cd ± 0.09	2.46 cd ± 0.09	4.89 cd ± 0.11
**T7**	0.00	1.06^c^ ± 0.01	2.92^g^ ± 0.07	3.93^g^ ± 0.12	6.56^g^ ± 0.12
**T8**	0.00	0.08^a^ ± 0.01	0.92^c^ ± 0.05	1.51^c^ ± 0.08	3.90^c^ ± 0.07
**T9**	0.00	0.09^a^ ± 0.01	0.83 cd ± 0.09	1.68 cd ± 0.03	3.96 cd ± 0.06
**T10**	0.00	0.06^c^ ± 0.01	1.51^de^ ± 0.03	2.29^de^ ± 0.04	5.55^de^ ± 0.08
**T11**	0.00	0.09^a^ ± 0.01	0.75^b^ ± 0.04	1.61^b^ ± 0.07	4.03^b^ ± 0.11
**T12**	0.00	0.08^a^ ± 0.01	0.82^a^ ± 0.03	1.55^a^ ± 0.16	4.14^a^ ± 0.10
**T13**	0.00	0.54^b^ ± 0.01	1.76^ab^ ± 0.05	2.52^ab^ ± 0.10	5.79^ab^ ± 0.08
**T14**	0.00	0.65^b^ ± 0.01	1.55^f^ ± 0.12	2.67^f^ ± 0.10	5.82^f^ ± 0.09
**T15**	0.00	0.51^e^ ± 0.01	1.46^de^ ± 0.05	2.99^de^ ± 0.49	5.56^de^ ± 0.10
**T16**	0.00	0.49^b^ ± 0.01	1.67^ef^ ± 0.12	2.73^ef^ ± 0.08	5.96^ef^ ± 0.12

Mean ±standard deviation (n = 3); rows with similar alphabet (on a particular storage week) designates that the means are significantly indifferent (p ≤ 0.05) according to DMRT

Fundamentally, the results documented in T1 to T16 revealed that, bioactive compounds have varying capability of compromising fungus membrane impermeability, spore propagation inhibition, which will results in depreciation of the fungus performance [104]. Curcuminoids’ is known for its ability to compromise of fungal cell stability. This results in severe outflow of the essential constituents of the cell, therefore exposing the cell to advance antifungal activities [105]. Additionally, saponins, alkaloids, and phenolic acids, which are present in most of the treatments, are highly effective in disrupting the enzymatic activities of fungi. These mechanisms will inhibit the assimilation of essential nutrients; obstruct the performance of the fungus, leading to a reduction in reproduction [103,106,107]. Saponins interrupt the structural integrity of fungal cells, cellular respiration, and the electron transport chain, resulting in membrane destabilization, metabolic crisis, cytolysis, and ultimately, fungal cell [108]. Furthermore, phenolic acids, due to their acidity nature, tend to lower the pH of the fungal cells intracellular constituents, as well as metals chelation. This can result in protein inactivation, ROS damage, and metabolic quiescence, which can significantly affect fungal cells activities [87]. Generally, alkaloids retard fungal cell homeostasis and survivability by disrupting cell membrane fluidity, DNA unwinding enzymes actions, and fat hydroperoxides [109]. Generally, data obtained from this investigation have confirmed that most plant derivatives tend to play an indispensable role in food safety and preservation.

#### 3.4.4. Isolated Fungal strains.

The results of the isolated fungal species from the fish specimens are presented in Table 9. Notably, the treatment plans significantly affected the survival and growth of individual fungal species in the fish tissues during the storage period (p ≤ 0.05). Though the identities of the fungal strains were not established by the molecular technique, the utilization of AFPA and PDA coupled with structural features during the clinical analysis usually assisted, in the tentative identification of *Aspergillus flavus* and *Aspergillus niger* isolates. Notably, the population of fungal strains was highest in the control unit compared to the enriched fish samples. At the eighth week of storage, the control group showed *Aspergillus spp. (black mold), Penicillium spp., Aspergillus spp. (flavus group),* and *Rhizopus spp.* counts of 84, 51, 99, and 85 CFU/g, respectively. Similarly, at the eighth week of storage, treated fish samples recorded *A. niger* ranges from 11 to 63 CFU/g, *Penicillium spp*. count that varied from 8 to 41 CFU/g, *A. flavus* population which was between 33 and 81 CFU/g, and *Rhizopus spp*. count which was between 11 and 72 CFU/g. Additionally, the dominance of *A. flavus* and *Rhizopus spp*. in most of the specimens depict the susceptibility the fish muscles to these fungal strains.

**Table 9:** The isolated fungi in the fish (x10^2^ CFU/g).

Treatment	*Aspergillus spp. (black mold)*		*Penicillum spp.*		*Aspergillus spp. (flavus group)*		*Rhizopus spp.*	
	Wk 0	Wk 8	Wk 0	Wk 8	Wk 0	Wk 8	Wk 0	Wk 8
**Control**	0.00	0.84^i^ ± 0.04	0.00	0.51^g^ ± 0.03	0.00	0.99^g^ ± 0.06	0.00	0.85^i^ ± 0.07
**T1**	0.00	0.63^h^ ± 0.02	0.00	0.41^f^ ± 0.02	0.00	0.81^f^ ± 0.02	0.00	0.72^h^ ± 0.02
**T2**	0.00	0.18^b^ ± 0.03	0.00	0.15^b^ ± 0.03	0.00	0.43^b^ ± 0.02	0.00	0.24^d^ ± 0.05
**T3**	0.00	0.24^c^ ± 0.03	0.00	0.13^ab^ ± 0.04	0.00	0.45^b^ ± 0.02	0.00	0.29^d^ ± 0.03
**T4**	0.00	0.49^g^ ± 0.02	0.00	0.39^e^ ± 0.02	0.00	0.73^e^ ± 0.03	0.00	0.42^g^ ± 0.03
**T5**	0.00	0.31^de^ ± 0.02	0.00	0.05^ab^ ± 0.02	0.00	0.35^a^ ± 0.03	0.00	0.17^bc^ ± 0.04
**T6**	0.00	0.33^e^ ± 0.02	0.00	0.08^bc^ ± 0.02	0.00	0.33^a^ ± 0.03	0.00	0.19^b^ ± 0.03
**T7**	0.00	0.47^g^ ± 0.03	0.00	0.33^d^ ± 0.03	0.00	0.71^e^ ± 0.04	0.00	0.44^g^ ± 0.02
**T8**	0.00	0.11^a^ ± 0.02	0.00	0.07^a^ ± 0.03	0.00	0.37^a^ ± 0.04	0.00	0.14^b^ ± 0.03
**T9**	0.00	0.12^a^ ± 0.02	0.00	0.08^a^ ± 0.03	0.00	0.39^a^ ± 0.02	0.00	0.19^c^ ± 0.02
**T10**	0.00	0.32^e^ ± 0.03	0.00	0.09^a^ ± 0.04	0.00	0.51^c^ ± 0.05	0.00	0.15^b^ ± 0.06
**T11**	0.00	0.12^a^ ± 0.03	0.00	0.08^a^ ± 0.03	0.00	0.37^a^ ± 0.02	0.00	0.11^a^ ± 0.03
**T12**	0.00	0.11^a^ ± 0.02	0.00	0.12^ab^ ± 0.02	0.00	0.36^a^ ± 0.05	0.00	0.17^bc^ ± 0.03
**T13**	0.00	0.35^ef^ ± 0.03	0.00	0.15^b^ ± 0.04	0.00	0.44^c^ ± 0.02	0.00	0.28^f^ ± 0.03
**T14**	0.00	0.32^e^ ± 0.03	0.00	0.17^b^ ± 0.04	0.00	0.67^d^ ± 0.02	0.00	0.39^f^ ± 0.03
**T15**	0.00	0.34^e^ ± 0.04	0.00	0.22^c^ ± 0.03	0.00	0.58^c^ ± 0.04	0.00	0.37^f^ ± 0.03
**T16**	0.00	0.38^f^ ± 0.04	0.00	0.15^b^ ± 0.05	0.00	0.66^d^ ± 0.05	0.00	0.33^e^ ± 0.02

Mean ±standard deviation (n = 3); rows with the same small alphabet for sample pathogen represent significant indifference (p ≤ 0.05) according to DMRT

Specifically, T8, T9, T11, and T12 exhibited the most potent antifungal activities, and this can be attributed to the mechanistic actions of the hybridized bio-additives, which were enhanced by the lemon extract acidity. The low pH lemon extract interferes with the fungal cell wall integrity, leading to greater osmotic pressure, and weakens the fungal resistance pathways [110]. This exposes the cell to secondary antifungal actions, by potent antifungal phytochemical compounds present in the other materials used for the hybridized treatments formulation. At the eight week, *Aspergillus spp. (black mold)* and *Rhizopus spp.* counts in the T8, T9, T11 and T12 experimental units, were smaller than 15 CFU/g and 20 CFU/g, respectively. Equally, T13, T14, T15, and T16 shown moderate antifungal activity; however, T1, T4, and T7 displayed very low antifungal effects. The antifungal effectiveness observed in this research, was similar to documented reports of these scholars [12,13,59], which stated that plant derivatives are potential antifungal agents in the fish industry.

The antifungal actions of the most potent treatments (T8, T9, T11, and T12), can be linked to the mechanistic actions of their basic phytochemical compounds. Gingerols, shogaols, zingerone, curcuminoids, turmerones, and phenolic acids, are abundant in ginger and turmeric rhizomes. These compounds have the ability of disrupting fungal cell mitochondrial physiology, as well as interfering with ATP production, hence resulting in the poor fungal growth rate and high mortality rate [111]. Gingerols belong to the lipophilic family, which has the ability to increase fungal permeability, and inhibit spore and ATP formation, eventually resulting in fungal cell lysis [112]. Zingerone hinders fungal proteins biosynthesis, leading to disruption of mycelial growth. This causes drastic reduction of fungal activities, in the fish muscles during storage. Furthermore, the antioxidant actions of the treatments help reduce oxidative stress, which enhances their antimicrobial properties, and consequently inhibits the fungal growth that can cause food deterioration [28,91].

Saponins have the ability to retard the binding of the fungal cell to ergosterol, an essential compound required by the microbes for active growth. This results in nutrient antagonism and poor spore formation, particularly in *Aspergillus spp., Penicillium spp*., and *Rhizopus spp.* [113]. Also, saponins cause the seepage of cytoplasm from *A. niger*, thereby retarding the mycelial growth [114]. It has been documented that saponins compromise the *Penicillium spp*. cell membrane performance and spore germination, which leads to impaired spore production and a high death rate [115]. Basically, phenolic acids interfere with *Aspergillus spp*. growth, by inhibiting the production of mycotoxin genes and disrupting the precursor pool [116]. Alkaloids have strong anti-*Rhizopus spp*. activity by weakening the hyphal membranes of the cells, causing excessive osmotic pressure around the cells and leading to cell failure [117].

#### 3.4.5. Aflatoxins profile of the fish samples.

The results of the aflatoxin levels in the fish samples are presented in Table 10. It was observed that at week 0, all treatment units and control groups recorded no detectable aflatoxins. However, at week eight, there was a substantial variation in aflatoxin concentrations among treatments, indicating the differential effectiveness of the various treatments. This study’s findings have depicted that, the enriched fish samples AFB₁ and AFB_2_ concentrations varied from 0.00 to 4.86 µg/kg, and 0.00 to 3.71 µg/kg, respectively. Then in the Control group, the AFB₁ and AFB₂ concentrations were 7.20 µg/kg and 5.69 µg/kg, respectively. Notably, T8 to T11 unveiled the most resilient anti-toxigenic effects, recording LOD value for AFB_1_ and AFB_2_ at the eight week. Also, T12 and T13 displayed good anti-aflatoxin activity, with the AFB_2_ concentration smaller than 0.95 µg/kg. On the contrary, T1, T4, and T7 exhibited relatively moderate anti-aflatoxins action. This ranking confirms that, the treatments contain numerous phytochemicals, having different proficiency of inhibiting aflatoxin production in dried fish muscles [118]. It is well documented that phenolic acids have an effective anti-aflatoxin production effect, by inhibiting the activities of aflatoxigenic fungal strains, and their aflatoxin binding affinity. This occurs through mechanisms such as destroying the cell membrane, mineral chelation, and inhibiting ROS and the aflatoxin gene cluster [116]. Alkaloids disrupt the ability of *Aspergillus flavus* and *Aspergillus parasiticus* to produce aflatoxins, by constraining their cells’ oxidative equilibrium, protein and ATP production, as well as free radical scavenging mechanisms of the fugal cells. Furthermore, curcuminoids are known for their high potency in interfering with aflatoxigenic fungal activity, by constraining mitochondrial respiration, ATP production. This will eventually trigger free radical damage, causing excessive cell degeneration, and will reduce the formation of aflatoxins by the fungal cells [119,104].

**Table 10:** The Aflatoxin levels in the fish samples (µg/kg).

Treatment	AFB_1_		AFB_2_	
	Week 0	Week 8	Week 0	Week 8
**Control**	LOD	7.20^d^ ± 0.16	LOD	5.69^h^ ± 0.16
**T1**	LOD	4.86^d^ ± 0.18	LOD	3.71^g^ ± 0.06
**T2**	LOD	2.33^a^ ± 0.16	LOD	2.12^bc^ ± 0.16
**T3**	LOD	2.47^a^ ± 0.05	LOD	2.09^b^ ± 0.21
**T4**	LOD	3.77^c^ ± 0.31	LOD	2.95^f^ ± 0.26
**T5**	LOD	2.97^a^ ± 0.05	LOD	2.47^e^ ± 0.30
**T6**	LOD	2.63^a^ ± 0.05	LOD	2.27^de^ ± 0.08
**T7**	LOD	4.03^c^ ± 0.01	LOD	2.97^f^ ± 0.42
**T8**	LOD	LOD	LOD	LOD
**T9**	LOD	LOD	LOD	LOD
**T10**	LOD	LOD	LOD	LOD
**T11**	LOD	LOD	LOD	LOD
**T12**	LOD	LOD	LOD	0.92^a^ ± 0.07
**T13**	LOD	LOD	LOD	0.77^a^ ± 0.17
**T14**	LOD	2.47^a^ ± 0.06	LOD	2.27 cd ± 0.21
**T15**	LOD	2.93^b^ ± 0.05	LOD	2.83^e^ ± 0.02
**T16**	LOD	2.90^b^ ± 0.06	LOD	2.60^de^ ± 0.05

Mean ±standard deviation (n = 3); in the same column, values having similar lowercase letters indicate a significant indifference (p ≤ 0.05) based on DMRT; LOD – Limit of Detection

Remarkably, the AFB_1_ and AFB_2_ levels documented this study, aligned with the conclusions reported by [120] and Tian et al. [121]. Tian et al. [121] further elaborated that the active bioactive compounds found in plant oils and extracts have strong anti-aflatoxin formation potential. Fascinatingly, despite the presence of AFB_1_ and AFB_2_ in most of the treated fish samples, their concentrations were within the 4 µg/kg. However, some of the treated outcomes (T1 and T7) had AFB_1_ levels that exceeded 4 µg/kg, but were lower than 10 µg/kg. In summary, the antimicrobial effectiveness of these bio-additives, mainly the active constituents of ginger and turmeric, projected greater efficacy in preventing the formation of aflatoxins in dried fish samples. This enhances their prospects as natural preservatives and nutrient enhancers, leading to higher food safety and improved nutrient quality of stored fish products.

### 3.5. Limitation of the study

This study did not conduct in vivo experimental frameworks involving trained human panels to evaluate the impact of the various treatments on the sensory properties of the fish samples. This is one of the major factors affecting consumers’ preferences; therefore, future studies should address this limitation.

## 4. Conclusion

Food spoilage and food-borne diseases have become major threats to food access and health security. This research concentrated on harnessing the antimicrobial and antioxidant properties of functional bioactive compounds in food safety, shelf life, and dietary value of fish during storage. Fish flesh was treated with lime extract (LE), ginger peel extract (GPE), turmeric peel extract (TPE), banana peel extract (PPE), ginger peel oil (GPO), turmeric peel oil (TPO), and banana peel oil (PPO), as well as their hybridized combinations, to enhance nutritional values and retard pathogenic growth during storage. The results established that, the treatments have significant impact on the fish muscle shelf life and nutritional quality (p ≤ 0.05). Throughout the eight-week storage period, the control sample recorded the maximum total fungal and bacterial loads; whereas, the enriched fish samples exhibited distinctly smaller fungal and bacterial populations. This study’s findings clearly identified that the hybridized treatment T8, exhibited the optimal nutrients retention ability, antimicrobial activity and anti-toxin formation; hence, the most effective formulation. Based on microorganisms’ isolates, it was observed that the treatments significantly inhibit *A. niger, A. flavus, Penicillium spp, R. species, Bacillus spp., Salmonella spp., Staphylococcus spp.*, and *Listeria spp.* performance [122,27,123,124]. This study has scientifically established that, hybridized plant-based derivatives can effectively lower aflatoxin concentrations, to levels recommended by the EU and WHO for human consumption. Though this study has proven that agricultural waste materials. can be harnessed to provide a potent fish preservative; hover, further research is recommended to evaluate the impact of these extracts and EOs, on the sensory characteristics and lipid oxidation rate of the processed fish muscles.
